# Detailed Structural Characterization of Arabinans and Galactans of 14 Apple Cultivars Before and After Cold Storage

**DOI:** 10.3389/fpls.2018.01451

**Published:** 2018-10-02

**Authors:** Daniel Wefers, Ramona Flörchinger, Mirko Bunzel

**Affiliations:** Department of Food Chemistry and Phytochemistry, Institute of Applied Biosciences, Karlsruhe Institute of Technology, Karlsruhe, Germany

**Keywords:** pectins, arabinans, galactans, dietary fiber, cell wall, NMR spectroscopy, HPAEC-PAD, enzymatic hydrolysis

## Abstract

Physiological and textural properties of apples are greatly influenced by both cultivar and structural composition of their pectic polysaccharides. In previous studies, it was demonstrated that neutral pectic side chains (arabinans and galactans) play a major role during fruit development and postharvest processes. However, these complex polymers have a high structural heterogeneity, and some structural elements such as side chain substituents and substitution of neighboring residues cannot be analyzed by using conventional analytical methods. Therefore, fine structures of arabinans and galactans were analyzed in 14 apple cultivars before and after storage. Besides conventional methods such as methylation analysis, profiling approaches based on enzymatic cleavage were applied to obtain detailed information on the neutral side chains of pectins. Structurally different, highly branched arabinans and linear β-1,4-linked galactans were detected in all cultivars. By using enzymatic profiling approaches, rare structural elements such as β-arabinofuranose and α-arabinopyranose residues were detected. In addition, the combination of all methods indicated structural differences with regard to ramification position or patterns. Cold storage resulted in decreased portions of branched arabinans. It was demonstrated that arabinan decomposition is independent of previously detected structural variations. In addition, analysis of *endo*-arabinanase hydrolysates demonstrated that β-arabinofuranose containing side chains are enriched after storage and may play a major role in postharvest processes. Analysis of *endo*-galactanase hydrolysates showed decreased portions of galactan-bound, terminal α-arabinopyranose units after storage. Therefore, these residues are most likely removed during postharvest galactan decomposition. The results of this study demonstrate the high complexity of neutral pectin side chains in apples and that pectic structural elements are differently prone to postharvest modifications.

## Introduction

Apples are among the most popular fruits worldwide, and many cultivars of different sensory properties are commercially available. Besides low molecular weight compounds, which greatly influence flavor and physiological properties, cell wall polymers are important constituents of apples. The major polymers in plant cell walls are non-starch polysaccharides, which are also the main components of dietary fiber. Consequently, the structural composition of plant cell wall polysaccharides influences the physiological properties of plant-based foods. In addition, previous studies on the relationship between cell wall polysaccharide composition and softening of apples suggested that this negative sensory perception correlates with a degradation of pectins (Tong et al., 1999; Nara et al., 2001; Pena and Carpita, 2004; Brummell, 2006; Prasanna et al., 2007; Billy et al., 2008; Goulao and Oliveira, 2008; Gwanpua et al., 2014; Ng et al., 2015; Winisdorffer et al., 2015; Gwanpua et al., 2016).

Besides cellulose, pectins are the most abundant cell wall polysaccharides in fruits and vegetables (Harris and Smith, 2006). Because of their structural complexity, pectins are divided into subgroups, which differ in the structure and/or substitution of the galacturonic acid containing backbone. Homogalacturonan is composed of α-1,4-linked D-galacturonic acid residues, which can be further methylated and acetylated (Voragen et al., 2009). Xylogalacturonan are also made up of a homogalacturonan backbone, but are substituted with xylose units at position *O*3. Although this group of pectic polysaccharides is often less important, it has been demonstrated to be abundant in apples (Schols et al., 1995; Zandleven et al., 2006). Rhamnogalacturonan I is the second most abundant group of pectic polysaccharides after homogalacturonan. These highly complex polysaccharides are composed of a backbone of alternating units of α-1,2-linked rhamnose units and α-1,4-linked galacturonic acid units. The rhamnose residues may be substituted with neutral side chains such as arabinans and galactans at position *O*4. Arabinans consist of a backbone of α-1,5-linked arabinofuranose units, which can be ramified at position *O*3 and/or position *O*2. Galactans are comprised of β-1,4-linked galactopyranose units, which were described to be partially ramified with galactopyranose and arabinofuranose units (Voragen et al., 2009). Recently, we demonstrated that β-1,3-linked arabinobiose side chains are abundant in some arabinans, and that terminal and internal α-arabinopyranose units are substantial constituents of some galactans (Wefers et al., 2014; 2015; Wefers and Bunzel, 2016a,b). Studies on the non-starch polysaccharides of apples demonstrated that type I rhamnogalacturonans with typically complex, branched arabinans and rather linear galactans are important cell wall constituents (Stevens and Selvendran, 1984; Massiot et al., 1994; Redgwell et al., 1997; Nara et al., 2001; Pena and Carpita, 2004; Wefers and Bunzel, 2016a). Several studies also indicated that both arabinan and galactan portions are decreased during ripening and storage of apples (Fischer et al., 1994; Massiot et al., 1996; Redgwell et al., 1997; Tong et al., 1999; Nara et al., 2001; Pena and Carpita, 2004; Gwanpua et al., 2014; Winisdorffer et al., 2015). This is most likely due to an elevated expression and activity of α-arabinofuranosidase and β-galactosidase during ripening and storage of apples (Goulao et al., 2007; Wei et al., 2010; Gwanpua et al., 2014). Thus, the structural composition of arabinans and galactans may significantly influence the extent of enzymatic hydrolysis and, therefore, postharvest modifications. However, arabinan and galactan fine structures in apples and the detailed structural composition of pectins of different apple cultivars after harvest and after storage are scarcely analyzed. The most detailed study to date was carried out by Pena and Carpita (2004), who investigated the cell wall composition of four apple cultivars after harvest and after 5 weeks of storage by using methylation analysis. Pre- and post-storage variations in the structural composition of the cell wall polysaccharides of the four apple cultivars were detected with degradation of branched arabinans being the predominant storage related modification. Nara et al. (2001) also observed a decrease in branched and terminal arabinose residues when two apple cultivars were analyzed by methylation analysis after harvest and after 8 weeks of storage. However, although methylation analysis provides a valuable overview of the polysaccharide structures, this method does not allow for a detection of some fine structural elements. Therefore, no information is available on the occurrence and modification of, for example, clusters of arabinan backbone substitution and β-arabinose-containing arabinan side chains. In addition, there is a lack of knowledge regarding abundance and potential plant-physiological significance of the recently established α-arabinopyranose residues in the galactan chains.

Consequently, this study focused on the detailed structural analysis of arabinans and galactans of 14 established and novel apple cultivars before and after storage. To obtain as much structural information on the pectic side chains as possible, recently developed profiling approaches (Wefers and Bunzel, 2016a,b) were applied besides well-established methods such as methylation analysis.

## Materials and Methods

### Chemicals

If not stated otherwise, all chemicals used were of “p.a.” grade or better and were purchased from VWR (Radnor, PA, United States), Sigma-Aldrich (Schnelldorf, Germany), or Carl Roth (Karlsruhe, Germany). Thermostable α-amylase (EC 3.2.1.1, from *Bacillus licheniformis*, 20,000–60,000 U/mL) was purchased from Sigma. Protease (EC 3.4.21.14, subtilisin A from *B. licheniformis*, 300 U/mL), amyloglucosidase (EC 3.2.1.3, from *Aspergillus niger*, 200 U/mL), *endo*-arabinanase (EC 3.2.1.99, from *A. niger*, 9 U/mg), and *endo*-galactanase (EC 3.2.1.89, from *A. niger*, 408 U/mg) were purchased from Megazyme (Bray, Ireland).

### Samples

All apple cultivars were grown at the Competence Center of DLR Rheinpfalz (Klein-Altendorf, Germany) and were kindly provided by the Department of Safety and Quality of Fruit and Vegetables, MRI Karlsruhe. Apples were harvested between August and September 2014, and the pulp of at least 20 fruits was pooled to one sample which was used for characterization. Cold storage was performed at 1°C for 8 months. However, only eight cultivars were usable after storage (**Table 1**). Further details about characteristics, harvesting, and storage of the apple samples were described previously (Eisenmann et al., 2016). Apples were peeled, and the pulp without seeds and apple-core was freeze-dried. The dry apple pulp was milled by using a Retsch PM100 ball mill (1 min, 600 rpm).

**Table 1:** Apple cultivars analyzed in this study.

Descriptor	Cultivar	Usable after storage
A	Galiwa	✓
B	Pinova Ewelina	✓
C	Elstar v. d. Zalm	✓
D	Ladina	×
E	Red Topaz	✓
F	PRI 037	×
G	Gemini	✓
H	Zari	×
I	PRI 010	×
J	Crimson Crisp	✓
K	Isaaq	✓
L	Allurel	✓
M	Natyra	×
N	Lubera	×

### Isolation of Non-starch Polysaccharides

Prior to all structural analyses, non-starch polysaccharides were isolated from the dried and milled apple pulp. Low molecular weight compounds such as sugars and organic acids were removed by washing three times with ethanol/water 80:20 (v/v). The residue was freeze-dried, and 5 g of the dried residue was resuspended in water and incubated with 100 μL of thermostable α-amylase at 95°C for 5 min. After cooling, 200 μL of amyloglucosidase was added and the suspension was incubated at 60°C for 10 min. Subsequently, enzymes were inactivated at 100°C for 5 min, and soluble non-starch polysaccharides were precipitated by adding four volumes of ethanol. The resulting non-starch polysaccharide preparation (consisting of insoluble and soluble polymers) was washed several times with ethanol/water 80:20 (v/v) and freeze-dried.

### Methylation Analysis

Glycosidic linkages of the non-starch polysaccharides were determined by methylation analysis as described previously (Nunes et al., 2008; Wefers and Bunzel, 2015). In brief, the samples were dissolved in DMSO and methylated by addition of freshly ground NaOH and methyl iodide. The methylated polysaccharides were extracted by using dichloromethane and evaporated to dryness. The methylation procedure was repeated once. Subsequently, 2 M TFA was added, and the samples were hydrolyzed at 121°C for 90 min. The acid was evaporated, and the partially methylated monosaccharides were reduced by using sodium borodeuteride. After terminating the reaction with glacial acetic acid, 1-methylimidazole and acetic anhydride were added for acetylation. The PMAAs were extracted into dichloromethane, washed, and residual water was removed by freezing overnight at -18°C. Identification of the PMAAs was carried out on a GC-MS system (GC-2010 Plus and GCMS-QP2010 SE, Shimadzu) as described previously (Wefers and Bunzel, 2015). Relative quantification of the PMAAs was carried out on a GC-FID system (GC-2010 Plus, Shimadzu) using the same conditions as for GC-MS analyses. Molar response factors according to Sweet et al. (1975) were used for semiquantitative analysis.

### Chromatographic Profiling of Arabinan and Galactan Oligosaccharides

To analyze structural details of arabinans and galactans, oligosaccharides were generated by using *endo*-arabinanase and *endo*-galactanase. These oligosaccharides were analyzed according to a recently established HPAEC-PAD profiling approach (Wefers and Bunzel, 2016a). Non-starch polysaccharides (5 mg) were suspended in 500 μL of water, and the suspension was incubated with *endo*-arabinanase (2 U/100 mg non-starch polysaccharides) for 24 h at 40°C to liberate the arabinan oligosaccharides. For an efficient hydrolysis of galactans, the samples were suspended in water (10 mg/mL), pretreated at 121°C for 40 min, and hydrolyzed with *endo*-galactanase (10 U/100 mg non-starch polysaccharides) for 24 h at 40°C. After inactivation of the enzymes, dilution, and addition of raffinose, the hydrolysates were analyzed by HPAEC-PAD on a CarboPac PA200 column (250 mm × 3 mm i.d., 5.5 μm particle size, Thermo Scientific Dionex). Oligosaccharide concentrations were semiquantitatively determined by using the relative response factors of the arabinan and galactan oligosaccharides to raffinose. Isolation of standard compounds, separation conditions, relative response factors, and further details of the method are described elsewhere (Wefers and Bunzel, 2016a).

### NMR Spectroscopic Profiling of Arabinan Structural Elements

An NMR spectroscopy-based profiling approach described by Wefers and Bunzel (2016b) was used to analyze the enzymatically liberated arabinan structural elements. Non-starch polysaccharides (60 mg) were suspended in 2.5 mL of water and extracted at 121°C for 40 min. Subsequently, *endo*-arabinanase (10 U/100 mg non-starch polysaccharides) was added, and the samples were hydrolyzed for 24 h at 40°C. After inactivation of the enzymes, unhydrolyzed material was removed by centrifugation, and 50 μL of D_2_O was added to a 450 μL aliquot of the hydrolysate. NMR spectroscopic analysis was performed on an Ascend 500 MHz NMR spectrometer (Bruker, Rheinstetten, Germany) equipped with a Prodigy cryoprobe. Volume integration of previously established HSQC marker signals was performed by using TopSpin 3.2 (Bruker). Subsequently, relative response factors of the marker signals were used for a semiquantitative determination of the of various arabinan structural elements. For further details on the method, the reader is referred to literature (Wefers and Bunzel, 2016b).

## Results and Discussion

For a detailed structural characterization of arabinans and galactans, non-starch polysaccharides were isolated from the pulp of the apple cultivars. The pulp of at least 20 fruits was pooled to exclude major variations between individual fruits. The isolation procedure was designed to maintain as much structural information as possible by applying short thermal treatments and enzymatic digestion steps, thus reducing thermally induced modifications. After enzymatic starch digestion using pure enzyme preparations, an ethanol precipitation was performed to combine all (ethanol insoluble) polysaccharides in one fraction. This approach was chosen because it allows for a better comparison of the arabinan and galactan structures between the cultivars without considering variations in solubility.

Besides detailed structural characterization, the apple pulp composition was analyzed by using several other analytical approaches. Because this paper focuses on the structural characterization of arabinans and galactans, these data will not be discussed in detail, and analytical data for all cultivars are given in the **Supplementary Material**: the method described by Prosky et al. (1988) was used to analyze the contents of insoluble and soluble non-starch polysaccharides in the pulp of the different apple cultivars. For an initial assessment of the non-starch polysaccharide composition before and after storage, the monosaccharide composition after Saeman hydrolysis as well as methanolysis in combination with TFA hydrolysis was analyzed. Overall, only minor differences between the cultivars were observed (**Supplementary Tables S1–S4**). Among other postharvest modifications of the monosaccharides portions, decreased portions of arabinose and galactose were clearly observable in the stored samples. This is in good agreement with a depletion of arabinan and galactan side chains, which was previously described to occur during apple storage (Fischer et al., 1994; Massiot et al., 1996; Tong et al., 1999; Nara et al., 2001; Pena and Carpita, 2004; Gwanpua et al., 2014; Winisdorffer et al., 2015).

### Methylation Analysis

To obtain detailed information about the overall structural composition of the non-starch polysaccharides before and after storage, methylation analysis was applied (see **Table 2**). The detected PMAAs suggested the presence of cellulose, xyloglucans, minor amounts of mannans and xylans, as well as highly branched arabinans and mainly linear β-1,4-linked galactans. The same PMAAs were detected in all cultivars before and after storage, and the main differences were observed for arabinan-and galactan-derived PMAAs. This is in good agreement with data from monosaccharide analysis (**Supplementary Tables S2–S4**) and previous studies (Pena and Carpita, 2004). However, comparably large portions of terminal galactose units (compared to 1,4-linked galactose units) most likely resulted from underestimation of 1,4-linked galactose units under the conditions used, which was observed previously (Wefers and Bunzel, 2016b). Although the decreased portions of 1,4-linked galactose units relative to both terminal and 1,3,6-linked galactose units indicate galactan degradation during storage, only the relative portions of arabinan-derived PMAAs yield reliable information on the structural composition and its alteration. For the interpretation of the data, it has to be considered that the relative PMAA ratios were analyzed. This implicates that, for example, increasing PMAA portions may be due to synthesis of specific structural elements or simply due to degradation of other structural elements. However, synthesis of pectic neutral side chains during storage seems less likely.

**Table 2:** Glycosidic linkages (mol%) of the non-starch polysaccharides of different apple cultivars before and after storage as determined by methylation analysis.

	A	B	C	D	E	F	G	H	I	J	K	L	M	N
t-Ara*f*	10.6	13.8	12.5	10.5	13.0	11.4	12.0	12.4	12.3	12.7	11.7	11.1	10.0	10.8
t-Ara*p*	0.7	0.8	0.5	0.5	0.5	0.6	0.5	0.6	0.6	0.7	0.5	0.6	0.8	0.5
1,5-Ara*f*	7.7	6.9	8.5	9.4	8.2	7.5	6.1	8.9	6.3	6.8	8.5	7.9	8.8	8.3
1,2,5-Ara*f*	0.9	1.4	0.6	1.6	1.1	1.0	0.6	0.6	0.7	0.4	1.1	0.7	1.4	1.0
1,3,5-Ara*f*	4.9	4.6	7.8	3.2	4.8	5.3	6.2	7.3	5.3	7.6	4.1	5.2	4.7	4.6
1,2,3,5-Ara*f*	2.9	4.0	2.1	3.0	3.2	2.7	2.3	1.1	2.6	1.9	2.9	2.2	3.5	2.3
t-Gal*p*	2.5	2.8	2.2	2.1	2.5	2.9	2.5	2.4	2.4	2.6	2.5	2.9	4.2	2.3
1,4-Gal*p*	2.9	3.1	2.8	2.1	2.5	4.1	2.9	3.2	3.6	2.6	2.5	5.1	3.8	1.9
1,6-Gal*p*	0.3	0.3	0.3	0.2	0.3	0.3	0.1	0.2	0.2	0.2	0.2	0.3	0.3	0.2
1,3,6-Gal*p*	0.7	0.7	0.5	0.4	0.6	0.4	0.5	0.5	0.7	0.4	0.4	0.9	1.4	0.9
t-Glc*p*	1.2	0.9	1.3	1.2	1.3	1.7	1.1	1.2	0.9	0.8	1.0	1.5	1.2	1.8
1,4-Glc*p*	40.0	36.1	36.9	38.2	36.7	38.4	39.4	35.3	38.2	37.4	40.2	37.5	34.8	39.1
1,4,6-Glc*p*	7.2	7.7	7.0	9.0	7.8	7.0	7.6	7.6	8.2	8.0	7.1	7.0	7.5	8.0
t-Man*p*	0.4	0.3	0.4	0.2	0.3	0.3	0.2	0.4	0.4	0.2	0.3	0.3	0.2	0.3
1,4-Man*p*	1.9	1.9	1.7	0.9	1.5	1.6	1.4	1.7	1.2	1.4	2.1	1.2	1.5	1.4
1,2-Rha*p*	0.5	0.4	0.4	0.5	0.4	0.4	0.4	0.4	0.4	0.3	0.5	0.3	0.4	0.4
1,2,4-Rha*p*	0.6	0.5	0.4	0.4	0.4	0.4	0.5	0.5	0.5	0.5	0.6	0.5	0.5	0.4
t-Xyl*p*	9.6	8.9	8.5	10.2	9.0	9.6	10.9	10.1	10.0	10.6	8.4	9.5	9.1	10.7
1,2-Xyl*p*	3.0	3.3	3.6	4.4	4.0	3.1	3.2	4.0	3.6	3.4	3.5	3.6	4.2	3.4
1,4-Xyl*p*	1.5	1.6	2.0	2.1	2.0	1.3	1.7	1.9	1.8	1.6	2.0	1.8	1.8	1.4
**After storage**											
t-Ara*f*	5.3	10.1	6.3		6.1		5.3			9.2	4.2	5.1		
t-Ara*p*	0.4	0.6	0.4		0.4		0.5			0.5	0.7	0.7		
1,5-Ara*f*	10.7	8.1	10.9		7.3		9.6			9.5	6.0	7.5		
1,2,5-Ara*f*	0.4	1.1	0.3		0.5		0.2			0.3	0.5	0.5		
1,3,5-Ara*f*	2.7	3.5	4.5		2.8		4.0			6.8	1.9	3.0		
1,2,3,5-Ara*f*	0.8	2.5	0.8		1.0		0.6			1.2	0.8	0.8		
t-Gal*p*	2.1	2.7	2.1		2.4		2.1			2.5	2.8	3.0		
1,4-Gal*p*	2.1	2.4	1.9		2.4		1.9			1.8	2.1	3.6		
1,6-Gal*p*	0.4	0.2	0.3		0.3		0.2			0.2	0.2	0.4		
1,3,6-Gal*p*	1.0	1.3	1.4		0.9		0.8			0.7	1.2	1.4		
t-Glc*p*	1.3	0.8	1.2		1.2		1.5			0.9	1.0	0.8		
1,4-Glc*p*	44.3	39.6	40.2		45.0		43.1			39.6	50.4	43.1		
1,4,6-Glc*p*	7.5	8.8	7.7		9.1		8.3			8.1	8.6	8.9		
t-Man*p*	0.3	0.2	0.3		0.3		0.2			0.2	0.2	0.2		
1,4-Man*p*	2.0	1.9	1.9		1.8		1.2			1.5	2.1	1.4		
1,2-Rha*p*	0.5	0.4	0.6		0.4		0.5			0.4	0.6	0.4		
1,2,4-Rha*p*	0.4	0.5	0.5		0.4		0.4			0.4	0.6	0.6		
t-Xyl*p*	10.2	10.2	9.4		11.4		11.1			10.2	11.5	13.2		
1,2-Xyl*p*	5.0	3.5	6.0		4.4		5.6			4.2	3.1	3.8		
1,4-Xyl*p*	2.5	1.7	3.4		2.0		3.0			1.9	1.6	1.8		

*All analyses were performed in duplicate (technical replicates, each sample was pooled from at least 20 fruits); relative half range uncertainties were mostly < 5%. A = Galiwa, B = Pinova Evelina, C = Elstar v. d. Zalm, D = Ladina, E = Red Topaz, F = PRI 037, G = Gemini, H = Zari, I = PRI 010, J = Crimson Crisp, K = Isaaq, L = Allurel, M = Natyra, N = Lubera, t = terminal, Ara = arabinose, Gal = galactose, Glc = glucose, Man = mannose, Rha = rhamnose, Xyl = xylose, *f* = furanose, *p* = pyranose. Numbers indicate the substituted positions of a sugar unit.*

For a better interpretation, the portions of arabinan-derived PMAAs were used to calculate the normalized relative appearance (**Table 3**). The less abundant terminal arabinopyranose units were not taken into account because they are most likely attached to β-1,4-linked galactans. All other arabinose-derived PMAAs can mainly be assigned to arabinans. 1,5-Linked arabinofuranose units are derived from the linear arabinan backbone, whereas 1,3,5-, 1,2,5-, and 1,2,3,5-linked arabinofuranose units represent backbone units that are substituted at position *O*3, *O*2, or at either position. Terminal arabinose units mainly represent side chains of the branched arabinan backbone. This is confirmed by the approximately equal appearance of terminal arabinose and PMAAs derived from branched backbone residues (the portion of 1,2,3,5-linked arabinofuranose units has to be doubled because two terminal arabinose units are attached).

**Table 3:** Arabinan composition (mol%) of the non-starch polysaccharides of different apple cultivars before and after storage as determined by methylation analysis.

	A	B	C	D	E	F	G	H	I	J	K	L	M	N
t-Ara*f*	39.3	44.8	39.6	37.9	43.0	41.1	44.2	41.0	45.1	43.2	41.4	40.9	35.3	39.9
1,5-Ara*f*	28.5	22.6	27.1	33.9	26.9	27.0	22.4	29.4	23.2	23.0	30.2	29.3	31.0	30.6
1,2,5-Ara*f*	3.4	4.6	1.9	5.9	3.7	3.4	2.3	1.9	2.6	1.5	3.9	2.7	4.8	3.7
1,3,5-Ara*f*	18.0	14.9	24.8	11.5	15.9	18.9	22.7	24.2	19.5	25.9	14.4	19.1	16.5	17.1
1,2,3,5-Ara*f*	10.8	13.1	6.5	10.8	10.6	9.6	8.4	3.5	9.6	6.5	10.1	8.1	12.5	8.7
**After storage**											
t-Ara*f*	26.6	40.0	27.7		34.5		27.0			34.3	31.3	30.3		
1,5-Ara*f*	53.3	32.1	47.9		41.2		48.6			35.1	44.9	44.5		
1,2,5-Ara*f*	2.2	4.3	1.2		2.6		1.0			1.2	4.0	2.7		
1,3,5-Ara*f*	13.7	13.8	19.7		16.0		20.4			25.1	14.0	17.6		
1,2,3,5-Ara*f*	4.1	9.8	3.5		5.6		3.1			4.3	5.8	4.9		

*All analyses were performed in duplicate (technical replicates, each sample was pooled from at least 20 fruits); relative half range uncertainties were mostly < 5%. A = Galiwa, B = Pinova Evelina, C = Elstar v. d. Zalm, D = Ladina, E = Red Topaz, F = PRI 037, G = Gemini, H = Zari, I = PRI 010, J = Crimson Crisp, K = Isaaq, L = Allurel, M = Natyra, N = Lubera, t = terminal, Ara = arabinose, *f* = furanose. Numbers indicate the substituted positions of a sugar unit.*

Before storage, mostly comparable portions of branched and linear, 1,5-linked arabinose units indicated rather complex arabinans in all cultivars. The arabinan backbone is preferably branched at position *O*3 or at both positions, *O*3 and *O*2, whereas *O*2 branched backbone residues are less prevalent. Although only minor differences were observed for the portions of linear 1,5-linked arabinose units among cultivars, the ramification patterns clearly differ. For example, cultivar C contains the highest portion of 1,3,5-linked arabinose units, but low portions of 1,2,5- and 1,2,3,5-linked arabinose units. For cultivars A and B, decreased portions of 1,3,5-linked arabinose units are accompanied by increased portions of 1,2,5- and 1,2,3,5-linked arabinose units. Consequently, clearly different ratios between 1,3,5-linked and 1,2,5-/1,2,3,5-linked arabinose units are obtained (3.0 for cultivar C, 1.3 for cultivar A, and 0.8 for cultivar B). Varying portions of these three PMAAs can also be observed for the other cultivars.

Significant relative changes were also observed for arabinan derived PMAAs after storage. All cultivars showed increased portions of the PMAAs derived from linear, 1,5-linked backbone units, whereas the portions of PMAAs derived from branched backbone units clearly decreased. Notably, all three types of substitution appear to be decreased for almost all cultivars, but differences in the overall decrease of branched backbone units can be observed. In addition, the PMAA ratios after storage still indicate inter-cultivar differences of the arabinan structures. However, roughly comparable ratios between 1,3,5-, 1,2,5-, and 1,2,3,5-linked arabinose units are observed for many cultivars before and after storage. These results demonstrate that different arabinan structures exist and also varying extents of postharvest modifications of these polymers occur in the apple cultivars. Modification of arabinan structures as predominant non-starch polysaccharide-related postharvest event is also in good agreement with results from previous studies, which suggested an α-arabinofuranosidase catalyzed debranching of the arabinan backbone during storage (Nara et al., 2001; Pena and Carpita, 2004; Goulao et al., 2007; Wei et al., 2010). The structural differences indicated by methylation analysis also emphasize the need for a more detailed structural analysis.

### Arabinan and Galactan Profiling After Enzymatic Cleavage

Combining the results from compositional analysis (**Supplementary Material**) and methylation analysis indicates that structural differences and storage-related changes of non-starch polysaccharides are mostly related to pectic polysaccharides. During storage, degradation and/or de-ramification of arabinans and galactans as neutral pectic side chains appears to be most prevalent. However, limited information with regard to substitution patterns and types of substituents of these complex polysaccharides is obtained from methylation analysis. In addition, some structural elements such as 1,2,3,5-substituted arabinofuranose units may be the result of partial undermethylation. Therefore, recently developed profiling methods based on enzymatic cleavage of arabinans and galactans were applied to analyze structural details of these polysaccharides (Wefers and Bunzel, 2016a,b). These approaches are based on cleavage of unsubstituted regions of the arabinan/galactan backbone by *endo*-arabinanase/*endo*-galactanase. The enzymatic hydrolysis results in the formation of di- and oligosaccharides as backbone fragments being diagnostic for either unbranched (linear) or branched regions. Most of these arabinan and galactan di-/oligosaccharides were isolated from different plant materials and characterized in detail (Wefers et al., 2014, 2015; Wefers and Bunzel, 2016a). Routine analysis of these di- and oligosaccharides can be performed by HPAEC-PAD and/or NMR spectroscopy. In both cases, a semiquantitative estimation of the liberated oligosaccharides/structural elements is performed by integrating the corresponding peaks/signals and considering their relative response. Due to the preservation of many structural elements, these profiling methods yield valuable structural information, which cannot be obtained from conventional methods. For example, substituents such as β-arabinofuranoses can be detected, and the presence of 1,2,3,5-linked arabinofuranose units can be unambiguously demonstrated.

The *endo*-galactanase hydrolysates were analyzed only by using HPAEC-PAD, because NMR spectroscopy detects the same dimeric end products of the enzymatic hydrolysis, but with lower sensitivity. For a detailed structural analysis of arabinans, the *endo*-arabinanase hydrolysates were analyzed by both approaches, NMR spectroscopy and HPAEC-PAD. It has to be considered that NMR spectroscopic analysis of the *endo*-arabinanase hydrolysates yields information about all solubilized arabinan structural elements, whereas HPAEC-PAD is limited to the analysis of known oligosaccharides (Wefers and Bunzel, 2016a,b). It was already demonstrated that the obtained results from both approaches are comparable to conventional methods such as methylation analysis, but that additional information about the arabinan fine structures can be obtained (Wefers and Bunzel, 2016a,b).

#### Chromatographic Profiling of Galactan Oligosaccharides

**FIGURE 1:**
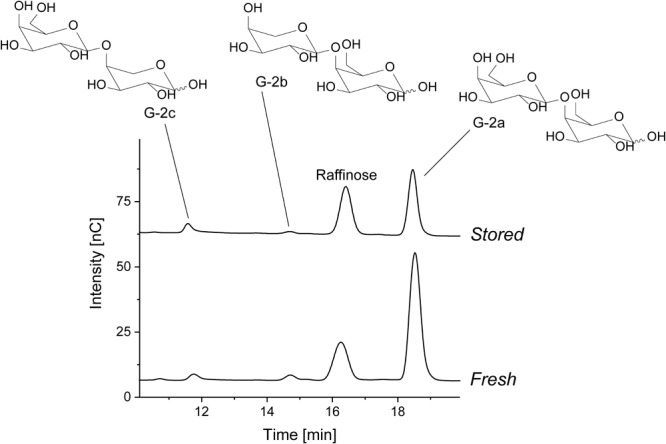
Structures of the liberated galactan oligosaccharides and HPAEC chromatograms of the *endo*-galactanase hydrolysate of cultivar A before and after storage. Raffinose was added to the hydrolysate as an internal standard.

Portions of enzymatically liberated galactan oligosaccharides as determined by HPAEC-PAD are shown in **Table 4**. An example for the *endo*-galactanase hydrolysates before and after storage of cultivar A is shown in **Figure 1**. Just as in all other cultivars, three galactan-derived disaccharides can be observed (structures are shown in **Figure 1**): the main product G-2a, which is derived from the *endo*-galactanase cleavage of the linear β-1,4-linked backbone, and two arabinopyranose containing disaccharides, G-2b and G-2c. Whereas G-2b mainly represents α-arabinopyranose residues that terminate galactan chains, G-2c is derived from internal, 1,4-linked α-arabinopyranose residues. Before storage, all cultivars only showed slight variations in the portions of β-1,4-linked galactobiose, but varying portions of the α-arabinopyranose containing oligosaccharides were observed. For example, cultivar B and cultivar C contained larger portions of G-2b than G-2c. In contrast, G-2c was present in slightly higher portions in cultivar A.

**Table 4:** Galactan oligosaccharide composition (mol%) of the non-starch polysaccharides of different apple cultivars before and after storage as determined by high performance anion exchange chromatography after *endo*-galactanase digestion.

	A	B	C	D	E	F	G	H	I	J	K	L	M	N
G-2a	92.1	91.6	93.6	88.7	91.1	91.3	91.0	90.7	90.7	89.3	88.5	92.1	91.6	88.2
G-2b	3.8	5.0	3.8	5.4	5.7	5.5	5.2	5.6	5.4	7.2	5.6	5.1	5.3	6.1
G-2c	4.1	3.4	2.6	5.9	3.2	3.2	3.8	3.7	4.0	3.6	6.0	2.8	3.1	5.8
**After storage**											
G-2a	86.2	88.8	93.5		92.5		83.2			89.0	82.3	93.5		
G-2b	2.8	3.1	1.8		2.2		2.2			1.9	2.0	2.7		
G-2c	11.0	8.2	4.7		5.4		14.6			9.1	15.8	3.8		

*The structures of the liberated oligosaccharides are shown in **Figure 1**. All analyses were performed in duplicate (technical replicates, each sample was pooled from at least 20 fruits); relative half range uncertainties were mostly < 5%. A = Galiwa, B = Pinova Evelina, C = Elstar v. d. Zalm, D = Ladina, E = Red Topaz, F = PRI 037, G = Gemini, H = Zari, I = PRI 010, J = Crimson Crisp, K = Isaaq, L = Allurel, M = Natyra, N = Lubera.*

Deviating galactan oligosaccharide portions were detected after storage: reduced portions of G-2a were liberated, for example, for cultivar A and B, whereas the portion of this oligosaccharide did not change over storage for cultivar C. In addition, G-2c was the dominant α-arabinopyranose containing oligosaccharide in all cultivars after storage whereas the portions of G-2b decreased. As stated above, the relative portions do not allow for a differentiation between an increase of one structural element or a decrease of another. However, considering the results from monosaccharide analysis and methylation analysis, it is likely that terminal α-arabinopyranose units (represented by G-2b) are removed during storage leading to an increased portion of internal α-arabinopyranose units (represented by G-2c). Therefore, these results indicate a degradation of the 1,4-linked galactan backbone during storage. Decreasing portions of terminal α-arabinopyranose units during storage suggest that these structural elements are not inhibitory to galactan degradation. This observation was also made when the chromatographic profiling approach was used to study postharvest ripening events of kiwifruits (Mack et al., 2017).

#### NMR-Spectroscopic Profiling of Arabinan Oligosaccharides

**FIGURE 2:**
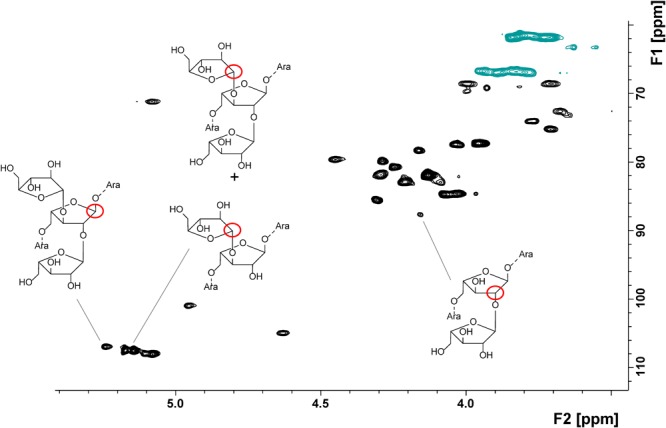
HSQC spectrum of the *endo*-arabinanase hydrolysate of cultivar B before storage as well as some of the detected structural elements.

NMR spectroscopic profiling allowed for the detection of various arabinan structural elements in the apple non-starch polysaccharides (**Table 5**). An exemplary HSQC spectrum with some of the detected structural elements is shown in **Figure 2**. Besides individual portions of structural elements, **Table 5** presents selected structural elements as sum parameters. The sum of terminal *O*5-bound arabinose (*O*5 indicates that this terminal residue is attached to position 5 of the second to last arabinose moiety in the chain) and 5-/1,5-linked arabinose units (5- and 1,5 indicates that the arabinose unit itself is linked in this/these position/s) may be helpful to interpret the data assuming that terminal *O*5-bound arabinose units mainly result from *endo*-arabinanase catalyzed cleavage of the linear arabinan backbone. In addition, the sum of terminal arabinose units either attached to the arabinan backbone at position *O*2 or *O*3 is listed.

**Table 5:** Arabinan composition (mol%) of the non-starch polysaccharides of different apple cultivars before and after storage as determined by two-dimensional NMR spectroscopy after *endo*-arabinanase digestion.

	A	B	C	D	E	F	G	H	I	J	K	L	M	N
5-/1,5-Ara*f*	19.5	16.6	5.8	19.1	15.9	17.1	16.0	20.1	16.9	20.1	10.5	17.5	18.2	19.9
t-*O*5-Ara*f*	15.1	13.5	14.0	14.7	15.7	14.8	14.0	13.6	13.6	9.8	15.6	13.6	15.3	14.8
t-*O*2-Ara*f*	15.4	18.4	16.1	17.6	22.4	15.5	13.5	10.7	15.8	11.5	14.6	16.1	19.1	13.8
t-*O*3-Ara*f*	21.5	24.3	26.4	24.0	21.4	25.2	28.0	25.9	24.9	27.8	26.7	25.2	19.8	23.0
Σ 5-/1,5-/t-*O*5-Ara*f*	34.5	30.0	19.8	33.8	31.5	31.9	29.9	33.6	30.5	29.9	26.1	31.1	33.5	34.7
Σ t-*O*3-/*O*2-Ara*f*	36.9	42.6	42.5	41.6	43.8	40.7	41.5	36.6	40.8	39.3	41.3	41.3	38.9	36.8
1,2,5-Ara*f*	3.8	3.9	1.3	5.6	2.5	3.2	1.1	1.3	2.7	1.2	2.8	2.4	3.9	3.0
1,3,5-Ara*f*	13.6	11.3	29.9	9.9	12.8	14.3	19.7	22.3	16.1	21.6	22.3	17.6	12.8	17.5
1,2,3,5-Ara*f*	9.6	11.1	6.5	9.1	9.3	9.8	7.8	6.2	10.0	7.0	7.5	7.6	10.9	7.9
t-β-Ara*f*	1.6	1.0	–	–	–	–	–	–	–	1.0	–	–	–	–
**After storage**											
5-/1,5-Ara*f*	34.5	23.7	27.9		25.8		27.4			23.0	30.2	31.1		
t-*O*5-Ara*f*	21.1	16.2	15.9		20.0		17.9			13.4	23.8	18.7		
t-*O*2-Ara*f*	7.6	14.8	5.7		11.9		7.2			10.4	10.9	10.7		
t-*O*3-Ara*f*	15.3	21.2	22.2		20.8		20.5			25.1	14.7	18.8		
Σ 5-/1,5-/t-*O*5-Ara*f*	55.5	40.0	43.8		45.7		45.4			36.4	54.0	49.8		
Σ t-*O*3-/t-*O*2-Ara*f*	22.9	36.1	27.9		32.6		27.7			35.5	25.6	29.5		
1,2,5-Ara*f*	3.5	4.0	2.0		2.8		2.1			1.5	4.6	–		
1,3,5-Ara*f*	10.2	10.5	22.4		12.6		17.9			21.4	12.4	17.8		
1,2,3,5-Ara*f*	3.6	8.5	3.9		6.3		4.9			5.2	3.4	2.9		
t-β-Ara*f*	4.3	1.0	–		–		1.9			–	–	–		

*An exemplary HSQC spectrum with some of the detected structural elements is shown in **Figure 2**. All analyses were performed in duplicate (technical replicates, each sample was pooled from at least 20 fruits); relative half range uncertainties were mostly < 10%. A = Galiwa, B = Pinova Evelina, C = Elstar v. d. Zalm, D = Ladina, E = Red Topaz, F = PRI 037, G = Gemini, H = Zari, I = PRI 010, J = Crimson Crisp, K = Isaaq, L = Allurel, M = Natyra, N = Lubera, t = terminal, Ara = arabinose, f = furanose. The portions (in %) of some structural elements are listed summarized and individually. Numbers indicate the substituted positions of a sugar unit and *O*2/*O*3/*O*5 indicates the position to which a terminal residue is attached.*

In agreement with methylation analysis, the NMR spectroscopic approach suggests varying ramification patterns of the arabinan backbone of the different cultivars. These differences are shown by considering the ratios between 1,3,5-Ara*f* to 1,2,5/1,2,3,5-Ara*f* units, which, e.g., decreased from cultivar C (3.8) to cultivar A (1.0) and cultivar B (0.8). After storage, lower portions of the structural elements 1,3,5- and 1,2,3,5-Ara*f* indicate removal of arabinan side chains. In addition, the ratios between 1,3,5-Ara*f* to 1,2,5/1,2,3,5-Ara*f* units showed the same trends as in the fresh samples. Thus, NMR spectroscopic analysis of the *endo*-arabinanase hydrolysates confirms the trends obtained from methylation analysis and also unambiguously confirms the presence of all structural elements. Furthermore, the NMR spectroscopic approach allowed for the detection of terminal β-arabinofuranose units in some cultivars before and/or after storage. For example, the portion of this structural element remains constant in cultivar B, whereas a relative increase after storage is detected for cultivar A. These results demonstrate that β-arabinofuranose units are part of apple arabinans and that they may have a distinct role during postharvest processes. However, although NMR spectroscopy allowed for the detection of this minor structural element and yielded valuable information on the arabinan composition, its inherent low sensitivity is generally less suitable for the analysis of minor structural elements (Wefers and Bunzel, 2016b). This can be overcome by analyzing the *endo*-arabinanase hydrolysates by HPAEC-PAD. This method also yields more information on the side chain composition or the substitution of neighboring backbone units.

#### Chromatographic Profiling of Arabinan Oligosaccharides

**FIGURE 3:**
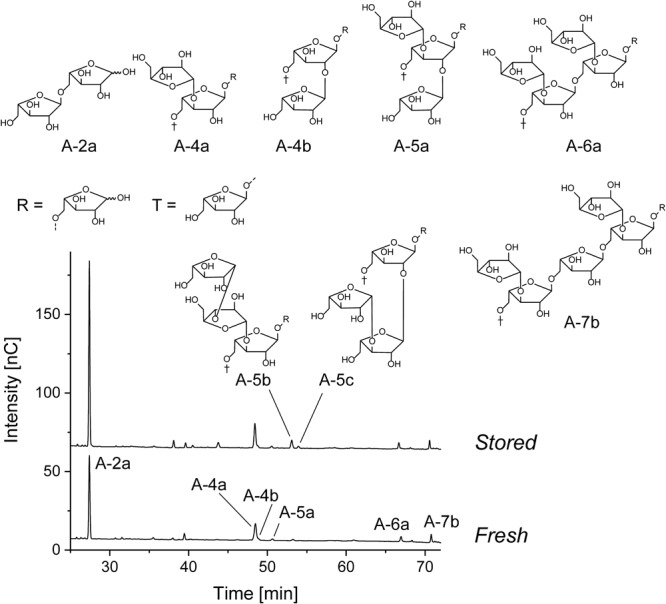
Structures of the liberated arabinan oligosaccharides and HPAEC chromatograms of the *endo*-arabinanase hydrolysate of cultivar C before and after storage.

The arabinan oligosaccharides portions that were obtained from the chromatographic profiling approach showed large variations among all cultivars (**Table 6**), and various branched arabinan oligosaccharides were detected besides the main hydrolysis product, arabinobiose, in the *endo*-arabinanase hydrolysates (**Figure 3**). The three oligosaccharides A-4a, A-4b, and A-5a (structures are shown in **Figure 3**) are each composed of three backbone arabinose units carrying arabinose side chains at position *O*3, *O*2, or both *O*3 and *O*2, respectively, representing the basic ramification patterns of the arabinan backbone. Among these three oligosaccharides, A-4a dominated in all cultivars. As mentioned above, several inter-cultivar differences were observed. For example, cultivars B and C showed comparable portions of A-4a, but cultivar B contained higher portions of A-4b and A-5a than both cultivars A and C. These results are in good agreement with the predominance of 1,3,5-linked arabinose units as detected by methylation analysis and NMR spectroscopy. However, some cultivars (e.g., cultivar A) showed a significantly lower overall ramification (determined by summarizing the portions of A-4a, A-4b, and A-5a) than other cultivars if compared to the results from methylation analysis and NMR spectroscopy. This variation could be derived from the fact that methylation analysis usually includes all polysaccharides in the sample, whereas NMR spectroscopy allows for the detection of all oligosaccharides solubilized by *endo*-arabinanase. In contrast, the chromatographic approach solely detects oligosaccharides with a degree of polymerization up to 7. Therefore, the decreased relative portions of branched, low molecular weight arabinan oligosaccharides indicate the presence of highly branched areas in cultivar A, resulting in branched oligo- or polysaccharides with a higher degree of polymerization after *endo*-arabinanase catalyzed digestion.

**Table 6:** Arabinan oligosaccharide composition (mol%) of the non-starch polysaccharides of different apple cultivars before and after storage as determined by high performance anion exchange chromatography after *endo*-arabinanase digestion.

	A	B	C	D	E	F	G	H	I	J	K	L	M	N
A-2a	85.9	68.7	71.5	85.1	73.6	83.7	69.2	80.1	73.0	63.8	81.7	85.7	84.0	80.6
A-4a	7.4	17.7	17.2	9.5	16.1	10.8	16.9	13.2	16.9	18.9	12.3	9.9	11.5	13.2
A-4b	0.3	3.0	1.5	1.9	1.6	1.2	–	0.7	–	1.9	0.7	0.6	0.5	0.5
A-5a	0.7	4.4	1.5	1.0	2.9	0.9	6.3	0.7	5.3	4.1	1.0	1.3	1.2	1.0
A-5b	3.8	–	1.2	–	–	–	1.0	–	–	1.2	–		–	0.5
A-5c	–	–	–	–	–	–	–	–	–	–	–	–	–	–
A-6a	0.9	2.5	3.7	0.9	2.5	1.6	2.6	2.9	1.8	4.4	2.0	1.1	1.1	2.1
A-7b	1.0	3.6	3.5	1.6	3.3	1.8	3.9	2.4	3.1	5.7	2.3	1.5	1.8	2.1
**After storage**											
A-2a	87.9	84.4	83.9		85.7		86.2			80.3	90.1	91.4		
A-4a	2.1	5.6	8.8		9.4		5.3			10.4	3.1	3.3		
A-4b	–	0.8	0.7		0.9		0.4			0.6	–	–		
A-5a	–	1.1	–		–		1.6			0.7	0.5	–		
A-5b	6.6	2.5	2.6		0.4		3.2			3.8	3.2	3.7		
A-5c	3.4	4.0	0.9		0.4		1.6			0.6	2.3	1.1		
A-6a	–	0.6	1.6		1.2		0.8			1.8	0.3	–		
A-7b	–	0.9	1.5		2.0		0.9			1.8	0.4	0.5		

*The structures of the liberated oligosaccharides are shown in **Figure 3**. All analyses were performed in duplicate (technical replicates, each sample was pooled from at least 20 fruits); relative half range uncertainties were mostly < 5%. A = Galiwa, B = Pinova Evelina, C = Elstar v. d. Zalm, D = Ladina, E = Red Topaz, F = PRI 037, G = Gemini, H = Zari, I = PRI 010, J = Crimson Crisp, K = Isaaq, L = Allurel, M = Natyra, N = Lubera.*

HPAEC-PAD oligosaccharide profiling also allowed for the detection of oligosaccharide A-5b in several cultivars, demonstrating the presence of complex, β-arabinobiose containing arabinan side chains. In addition, the oligosaccharides A-6a and A-7b, which represent highly branched areas of the arabinan backbone were detected in all cultivars. The portions of these compounds (A-6a and A-7b) indicate that substitution of two neighboring backbone arabinose units is more prevalent in some cultivars such as cultivar C or J. However, as mentioned above, the substitution of more than two neighboring backbone units is plausible if the results from methylation analysis and NMR spectroscopy are considered.

After storage, the portion of A-2a (α-1,5-linked arabinobiose) increased whereas the portions of the branched oligosaccharides A-4a, A-4b, and A-5a decreased. Again, this indicates debranching of the arabinan backbone, independently of the ramification position. These observations are in good agreement with data from methylation analysis and NMR spectroscopy, which also demonstrated decreasing portions of 1,3,5-linked, 1,2,3,5-linked, and (in case of methylation analysis) 1,2,5-linked arabinose units. Notably, portions of the highly branched oligosaccharides A-6a and A-7b are also reduced after storage, indicating that substitution of neighboring backbone units does not impede removal of α-arabinose side chains. Portions of the liberated branched oligosaccharides after storage reveal some differences between the cultivars: For example, the *endo*-arabinanase hydrolysate of cultivar J contained the highest overall portion of the α-arabinose branched oligosaccharides A-4a, A-4b, A-5a, A-6a, and A-7b before and after storage. Other cultivars such as cultivar A showed low portions of these oligosaccharides before storage and only contained small portions of A-4a after storage (the other oligosaccharides were below the limit of quantitation). Therefore, the portion of branched oligosaccharides before storage correlates with the oligosaccharide portions after storage.

In contrast to all other branched oligosaccharides, portions of the oligosaccharides A-5b and A-5c, containing β-arabinofuranose side chains, increased in all three cultivars after storage. Thus, the increase of β-arabinofuranose units, as also detected for some cultivars by using the NMR spectroscopic profiling approach, is most likely due to the relative increase of the β-arabinofuranose containing side chains. Decreasing arabinose contents and removal of α-linked arabinose units during storage suggest an α-arabinofuranosidase mediated degradation of the arabinan side chains. Therefore, it seems likely that β-arabinofuranose containing side chains as found in A-5b and A-5c are not formed, but simply retained during storage.

## Conclusion

The structural characterization of arabinans and galactans in apple pulp demonstrated the presence of highly branched arabinans and rather linear galactans. Some distinct variations were observed between the cultivars. In-depth structural analyses after 8 months of cold storage suggested a degradation of galactan side chains, which was not impeded by the occurrence of terminal α-arabinopyranose units. However, removal of arabinan side chains was found to be the predominant postharvest modification. This was found to be largely independent of the arabinan ramification (degree of substitution, substitution of neighboring backbone residues); α-arabinofuranose side chains bound to single or neighboring arabinan backbone units are removed during storage. However, β-arabinofuranose containing side chains may play a particular role in postharvest modifications because they were of higher relative abundance after storage. The present study also demonstrates the importance of analyzing the fine structures of complex polymers such as arabinans and galactans, which can only be achieved by some more advanced analytical approaches.

## Author Contributions

DW and MB designed the research and wrote the manuscript. RF conducted the experiments. DW and RF analyzed the data and results. All authors approved the final version of the manuscript.

## Conflict of Interest Statement

The authors declare that the research was conducted in the absence of any commercial or financial relationships that could be construed as a potential conflict of interest.

## Abbreviations

HPAEC-PAD: high performance anion exchange chromatography with pulsed amperometric detection
PMAA(s): partially methylated alditol acetate(s)
TFA: trifluoroacetic acid
