# Supplementary material for: Detailed Structural Characterization of Arabinans and Galactans of 14 Apple Cultivars Before and After Cold Storage
Source: Front Plant Sci. 2018 Oct 2;9:1451. doi: 10.3389/fpls.2018.01451 (PMC6175989; doi:10.3389/fpls.2018.01451)
Supplement: Supplementary file 1 [file Data_Sheet_1.pdf]

Supplementary Material

Detailed structural characterization of arabinans and galactans of 14 apple cultivars before and after cold storage

Daniel Wefers[1*], Ramona Flörchinger[1], Mirko Bunzel[1]

*** Correspondence:** Daniel Wefers: daniel.wefers@kit.edu

1 Supplementary Methods

1.1 Determination of the non-starch polysaccharides contents

Non-starch polysaccharide contents were determined according to the dietary fiber method described by Prosky et al. (1988). Briefly, 1 g of untreated, milled apple flour was sequentially digested with 100 µL of thermostable α-amylase, 100 µL of protease, and 200 µL of amyloglucosidase. Insoluble dietary fiber/non-starch polysaccharides were recovered by filtration and soluble polysaccharides were precipitated from the supernatant by adding of 4 volumes of ethanol. The insoluble and soluble dietary fiber/non-starch polysaccharide contents were corrected for protein (determined as NH_4^+ according to Willis et al. (1996) after Kjeldahl digestion, nitrogen conversion factor of 6.25) and ash (incineration for 5 h at 525 °C). All analyses were performed in duplicate (technical replicates).

1.2 Monosaccharide analysis after acidic hydrolysis

Sulfuric acid hydrolysis of the apple non-starch polysaccharides was performed according to Saeman et al. (1945) The samples (10 mg) were swollen in 150 µL of 12 M sulfuric acid for 2.5 h, diluted with 975 µL of water, and hydrolyzed for 3 h at 100°C. After filtration and dilution, the hydrolysate was analyzed by high performance anion exchange chromatography with pulsed amperometric detection (HPAEC-PAD) using an ICS-5000 system (Thermo Scientific Dionex, Sunnyvale, CA) equipped with a CarboPac PA20 column (150 mm x 3 mm i.d., 6.5 µm particle size, Thermo Scientific Dionex). A flow rate of 0.4 mL/min and a gradient composed of (A) bidistilled water, (B) 0.1 M sodium hydroxide, and (C) 0.1 M sodium hydroxide + 0.2 M sodium acetate were used at 25°C: Before every run, the column was rinsed with 100% B for 10 min and equilibrated for 20 min with 90% A and 10% B. After injection, the following gradient was applied: 0-1.5 min, from 90% A and 10% B to 96% A and 4% B; 1.5-22 min, isocratic, 96% A and 4% B; 22-32 min, from 96% A and 4% B to 100% B; 32-42 min, isocratic, 100% C. Methanolysis in combination with trifluoroacetic acid (TFA) hydrolysis was carried out as described previously (De Ruiter et al., 1992; Wefers and Bunzel, 2015). Briefly, 2 mL of methanolic HCl (1.25 M) was added to the sample (10 mg), and methanolysis was performed for 16 h at 80°C. An aliquot was evaporated, hydrolyzed with 2 M TFA (500 µL) for 1 h, evaporated to dryness, and the monosaccharide composition was analyzed by HPAEC-PAD as described above. In addition, the colorimetric approach described by Blumenkrantz and Asboe-Hansen (1973) was used as an additional method to determine the galacturonic acid content of the apple non-starch polysaccharides.

References

Blumenkrantz, N., and Asboe-Hansen, G. (1973). New method for quantitative determination of uronic acids. *Anal. Biochem.* 54**,** 484-489.

De Ruiter, G.A., Schols, H.A., Voragen, A.G.J., and Rombouts, F.M. (1992). Carbohydrate analysis of water-soluble uronic acid-containing polysaccharides with high-performance anion-exchange chromatography using methanolysis combined with TFA hydrolysis is superior to four other methods. *Anal. Biochem.* 207**,** 176-185.

Prosky, L., Asp, N.G., Schweizer, T.F., Devries, J.W., and Furda, I. (1988). Determination of insoluble, soluble, and total dietary fiber in foods and food products: Interlaboratory study. *J. AOAC Int.* 71**,** 1017-1023.

Saeman, J.F., Bubl, J.L., and Harris, E.E. (1945). Quantitative saccharification of wood and cellulose. *Ind. Eng. Chem., Anal. Ed.* 17**,** 35-37.

Wefers, D., and Bunzel, M. (2015). Characterization of dietary fiber polysaccharides from dehulled, common buckwheat (*Fagopyrum esculentum*) seeds. *Cereal Chem.* 92**,** 598-603.

Willis, R.B., Montgomery, M.E., and Allen, P.R. (1996). Improved method for manual, colorimetry determination of total Kjeldahl nitrogen using salicylate. *J. Agric. Food Chem.* 44**,** 1804-1807.

2 Supplementary Tables

Supplementary Table 1: Insoluble and soluble non-starch polysaccharide (NSP) contents (g/100 g dry matter) of different apple cultivars before and after storage.

		Insoluble NSP	R/2	Soluble NSP	R/2
Galiwa	*fresh*	6.7	0.1	4.2	0.2
	stored	7.0	0.0	5.6	1.4
Pinova Evelina	*fresh*	9.0	0.2	4.9	0.4
	stored	8.8	0.1	5.2	0.7
Elstar v. d. Zalm	*fresh*	7.0	0.0	4.8	1.0
	stored	6.8	0.1	4.1	0.5
Red Topaz	*fresh*	6.9	0.1	7.6	1.1
	stored	6.7	0.1	4.4	0.5
PRI 037	*fresh*	9.5	0.1	3.7	0.0
	stored	-	-	-	-
Gemini	*fresh*	8.9	0.0	5.4	0.5
	stored	8.5	0.1	4.5	0.3
Zari	*fresh*	6.6	0.1	3.9	0.6
	stored	-	-	-	-
PRI 010	*fresh*	7.4	0.1	6.1	0.1
	stored	-	-	-	-
Crimson Crisp	*fresh*	9.3	0.0	5.3	0.2
	stored	9.8	0.1	4.9	1.0
Isaaq	*fresh*	8.7	0.0	3.3	0.3
	stored	9.6	0.1	5.7	1.4
Allurel	*fresh*	6.9	0.1	2.9	0.1
	stored	6.9	0.4	3.3	0.2
Natyra	*fresh*	8.3	0.0	2.2	0.5
	stored	-	-	-	-
Lubera	*fresh*	10.9	0.3	4.9	1.2
	stored	-	-	-	-

All analyses were performed in duplicate (technical replicates); relative half range uncertainties were mostly < 5 %.

Supplementary Table 2: Monosaccharide composition (mol%) of the non-starch polysaccharides of different apple cultivars before and after storage. The monosaccharide composition was determined by high performance anion exchange chromatography after H_2SO_4 hydrolysis.

	**A**	**B**	**C**	**D**	**E**	**F**	**G**	**H**	**I**	**J**	**K**	**L**	**M**	**N**
Fucose	1.7	1.5	1.6	2.0	1.7	1.5	1.8	2.0	1.8	1.8	1.7	1.6	1.6	1.9
Rhamnose	1.5	1.4	1.8	1.7	1.4	1.5	1.6	1.7	1.4	1.6	1.8	1.7	1.6	1.7
Arabinose	16.8	19.3	18.4	17.0	17.1	15.6	18.5	18.9	17.7	19.3	17.8	17.4	16.5	16.7
Galactose	8.7	10.9	9.7	7.2	12.3	14.3	9.0	10.2	12.0	9.6	8.2	16.4	14.7	7.0
Glucose	38.1	36.2	38.2	43.3	37.5	36.4	37.8	35.1	36.8	38.0	38.9	34.4	36.3	39.6
Xylose	10.6	9.6	10.2	12.4	10.4	10.0	11.8	11.5	11.1	12.1	10.4	10.2	10.2	11.6
Mannose	7.4	6.7	6.1	4.2	5.2	5.9	6.6	7.5	5.9	5.0	5.9	4.5	4.8	5.8
Galacturonic acid	15.2	14.5	14.0	12.3	14.4	14.7	13.0	13.1	13.3	12.5	15.3	13.7	14.2	15.7
Glucuronic acid	tr	tr	tr	tr	tr	tr	tr	tr	tr	tr	tr	tr	tr	tr
**After storage**														
Fucose	2.3	2.0	2.5		2.6		2.2			1.9	2.7	2.3		
Rhamnose	1.4	1.3	1.6		1.2		1.5			1.3	2.6	2.4		
Arabinose	11.3	16.0	11.4		10.4		10.5			16.0	11.1	12.5		
Galactose	7.4	8.6	7.9		8.7		7.2			7.7	8.1	12.6		
Glucose	41.1	36.9	41.0		41.4		42.6			38.8	45.1	41.2		
Xylose	10.3	9.8	11.1		10.7		11.4			10.7	13.9	13.8		
Mannose	5.6	6.0	6.2		5.7		5.0			5.3	4.8	3.8		
Galacturonic acid	20.4	19.9	18.3		19.7		19.5			18.2	11.6	11.4		
Glucuronic acid	tr	tr	tr		tr		tr			tr	tr	tr		

All analyses were performed in duplicate (technical replicates); relative half range uncertainties were mostly < 10 %. A = Galiwa, B = Pinova Evelina, C = Elstar v. d. Zalm, D = Ladina, E = Red Topaz, F = PRI 037, G = Gemini, H = Zari, I = PRI 010, J = Crimson Crisp, K = Isaaq, L = Allurel, M = Natyra, N = Lubera, tr = traces.

Supplementary Table 3: Galacturonic acid contents (g/100 g dry matter) of the non-starch polysaccharides of different apple cultivars before (upper line) and after storage (lower line).

	**Galacturonic acid content**	**R/2**
Galiwa	23.2	0.5
	29.6	0.8
Pinova Evelina	25.6	1.1
	32.1	1.4
Elstar v. d. Zalm	24.1	0.2
	30.4	0.9
Ladina	22.2	0.1
	-	-
Red Topaz	26.8	0.5
	32.5	1.1
PRI 037	21.7	0.5
	-	-
Gemini	26.6	0.1
	30.1	0.6
Zari	25.5	1.5
	-	-
PRI 010	24.7	0.1
	-	-
Crimson Crisp	27.9	0.4
	28.2	0.7
Isaaq	28.1	0.0
	28.8	0.6
Allurel	26.1	0.8
	20.0	0.2
Natyra	22.8	0.7
	-	-
Lubera	23.4	0.4
	-	-

All analyses were performed in duplicate (technical replicates); relative half range uncertainties were mostly < 5 %.

Supplementary Table 4: Monosaccharide composition (mol%) of the non-starch polysaccharides of different apple cultivars before and after storage. The monosaccharide composition was determined by high performance anion exchange chromatography after methanolysis and trifluoroacetic acid hydrolysis.

	A	B	C	D	E	F	G	H	I	J	K	L	M	N
Fucose	2.6	2.1	2.3	2.8	2.3	2.0	2.4	2.5	2.6	2.5	2.2	1.9	1.9	2.7
Rhamnose	4.2	3.6	4.2	4.1	3.5	3.6	4.0	4.2	3.5	4.4	4.5	3.6	3.6	4.3
Arabinose	32.4	34.1	33.3	30.3	31.4	28.9	32.7	32.3	30.2	31.3	34.0	28.7	28.1	32.5
Galactose	15.8	20.4	17.9	12.8	22.7	24.8	15.8	17.2	21.0	16.2	14.7	26.1	24.8	12.6
Glucose	4.4	4.1	3.8	8.0	4.5	3.4	4.3	3.4	3.6	3.6	3.5	3.5	4.4	3.7
Xylose	14.7	13.7	14.1	16.6	13.4	13.0	15.4	14.7	14.0	14.3	14.7	13.4	13.6	16.6
Mannose	5.3	4.7	6.4	3.9	4.3	4.9	4.7	5.1	4.7	4.9	4.1	3.8	3.8	4.3
Galacturonic acid	19.9	16.6	17.2	21.0	17.2	18.6	20.8	20.5	19.9	23.0	22.4	18.6	19.3	22.6
Glucuronic acid	1.1	0.6	0.7	0.7	0.8	0.7	tr	tr	0.7	tr	tr	0.7	0.4	0.7
**After storage**														
Fucose	3.9	3.2	3.5		4.0		3.7			3.6	4.1	3.9		
Rhamnose	6.3	5.1	6.6		5.6		5.8			5.2	6.9	6.0		
Arabinose	24.9	31.5	24.4		22.2		22.4			30.8	20.2	21.1		
Galactose	14.7	16.1	15.5		16.4		13.7			14.4	14.3	21.6		
Glucose	5.9	5.5	5.6		6.4		5.2			4.8	5.1	4.7		
Xylose	18.7	16.5	18.2		19.3		20.7			18.0	19.4	18.3		
Mannose	3.9	3.5	4.3		3.9		3.5			3.1	3.6	2.7		
Galacturonic acid	21.5	18.6	21.7		22.3		24.3			19.6	25.9	21.2		
Glucuronic acid	tr	tr	tr		tr		0.8			0.7	0.8	0.8		

All analyses were performed in duplicate (technical replicates); relative half range uncertainties were mostly < 10 %. A = Galiwa, B = Pinova Evelina, C = Elstar v. d. Zalm, D = Ladina, E = Red Topaz, F = PRI 037, G = Gemini, H = Zari, I = PRI 010, J = Crimson Crisp, K = Isaaq, L = Allurel, M = Natyra, N = Lubera, tr = traces.
